# The Development of a One-Step PCR Assay for Rapid Detection of an Attenuated Vaccine Strain of Duck Hepatitis Virus Type 3 in Korea

**DOI:** 10.3390/vetsci12010008

**Published:** 2024-12-29

**Authors:** Cheng-Dong Yu, Jong-Yeol Park, Sang-Won Kim, Yu-Ri Choi, Se-Yeoun Cha, Hyung-Kwan Jang, Min Kang, Bai Wei

**Affiliations:** 1 Department of Avian Diseases, College of Veterinary Medicine and Center for Avian Disease, Jeonbuk National University, Iksan 54596, Republic of Korea; yuchengdong@naver.com (C.-D.Y.); jyp0410@jbnu.ac.kr (J.-Y.P.);; 2 Bio Disease Control (BIOD) Co., Ltd., Iksan 54596, Republic of Korea

**Keywords:** duck hepatitis A virus type 3, differentiating infected from vaccinated animals, mismatch amplification mutation assay, rapid diagnostic

## Abstract

Duck hepatitis A virus type 3 (DHAV-3) is a viral pathogen that causes acute, high-mortality hepatitis in ducklings, and vaccination with attenuated live vaccines is currently the main preventive measure against it. However, differentiating infected from vaccinated animals (DIVA) is crucial for clinical diagnosis and effective disease control. This study aimed to develop a rapid mismatch amplification mutation assay PCR (MAMA-PCR) diagnostic method to simultaneously detect and differentiate between wild-type and vaccine strains. The method was specifically designed to target the critical single-nucleotide polymorphism (SNP) site (T→C at position 1143 in the VP0 gene) unique to the Korean vaccine strain AP04203-P100. MAMA-PCR demonstrated high sensitivity and specificity, with detection limits as low as 10^2.4^ ELD_50_/mL for wild strains and 10^0.5^ ELD_50_/mL for vaccine strains, and showed no cross-reactivity with 11 other common duck pathogens. The clinical sample results were completely consistent with those obtained using nested PCR detection and gold-standard sequencing. In summary, we successfully developed a rapid, one-step MAMA-PCR method that is more suitable for clinical diagnosis than traditional sequencing methods.

## Simple Summary

Duck hepatitis A virus type 3 (DHAV-3) is a viral pathogen that can cause acute hepatitis characterized by rapid onset and high mortality in ducklings. Vaccination is the primary strategy for DHAV-3 prevention; live attenuated vaccines are widely used due to their strong immunogenicity and quick onset of action. Thus, differentiating infected from vaccinated animals (DIVA) is crucial for monitoring DHAV-3 infection and for achieving effective disease control. The only method currently available to differentiate wild-type and vaccine strains involves sequencing followed by analysis of specific loci, which is complex, time-consuming, and costly, limiting its clinical applicability. This study developed a one-step mismatch amplification mutation assay PCR (MAMA-PCR) targeting specific single-nucleotide polymorphisms (SNPs) identified during live attenuated vaccine development, enabling the simultaneous detection and differentiation of DHAV-3 wild-type and vaccine strains. This approach provides a rapid and efficient tool for the clinical diagnosis of DHAV-3 infections.

## 1. Introduction

Duck hepatitis A virus (DHAV) is a critical pathogen in the duck industry, causing high mortality and economic losses, particularly in young ducklings [1]. Based on phylogenetic analyses and virus neutralization tests, DHAV is divided into three serotypes: DHAV type 1 (DHAV-1), the most widespread serotype; DHAV type 3 (DHAV-3), a novel serotype that has emerged in South Korea; and DHAV type 2 (DHAV-2), which is geographically restricted to Taiwan and India [2,3,4,5]. DHAV-3 has surpassed DHAV-1 in prevalence across countries including South Korea, China, Vietnam, and Egypt [6,7,8,9], necessitating effective control measures. The primary preventive strategy is vaccination, and live attenuated vaccines are widely used due to their rapid induction of immunity and ease of administration [10,11,12]. Since live vaccines are largely used in the duck industry throughout the world, differentiating between the wild-type and vaccine strains is crucial for monitoring DHAV-3 infection [12,13].

The DHAV genome consists of a single long open reading frame (ORF) that encodes a polyprotein, which is subsequently cleaved into three structural proteins: VP0, VP1, and VP3 [14]. Of these, VP1 is a structural protein with potential antigenic properties, containing epitopes that are recognized by both B and T cells, playing a key role in initiating an immune response [15]. In addition, substitutions in the VP1, as well as the VP0 and VP3 regions, may affect the virulence or facilitate immune escape mechanisms [16]. The current methods for differentiating vaccine strains from wild-type strains of DHAV-3 rely on sequencing specific genomic regions [17]. However, these approaches are time-consuming, costly, and not feasible for rapid field diagnostics. To address this issue, the development of a faster, more accessible diagnostic tool is imperative. During the attenuation process of the AP-04203-P100 vaccine strain, several key single-nucleotide polymorphisms (SNPs) were identified as responsible for its reduced virulence [10]. These SNPs provide the basis for a diagnostic tool for differentiating between the wild-type and vaccine strains. Mismatch amplification mutation assay PCR (MAMA-PCR) is a promising method that uses SNP-specific primers to selectively amplify target sequences, allowing for precise differentiation between strains [18,19]. MAMA-PCR is not only straightforward to design but also inexpensive and suitable for use in standard laboratories, making it ideal for rapid detection [18,20]. In comparison to traditional methods, MAMA-PCR offers significant advantages in terms of speed and practicality, making it more accessible for routine diagnostics in both clinical and field settings. Furthermore, it provides a faster turnaround time, which is crucial for early detection and timely intervention, particularly in outbreak situations.

This study presents a novel application of MAMA-PCR for rapid and accurate differentiation between the DHAV-3 vaccine strain and wild-type strain. By targeting specific SNPs identified during vaccine development, this method offers a more practical and cost-effective alternative to sequencing, supporting both clinical diagnostics and the ongoing surveillance of DHAV-3 vaccine use. This method could be applied clinically to differentiate DHAV-3-infected ducks from vaccinated ducks, reducing the risk of economic losses due to misdiagnosis or delayed diagnosis and providing reliable support for ongoing safety and efficacy monitoring of the DHAV-3 vaccine in South Korea.

## 2. Materials and Methods

### 2.1. Virus Strains

The virus strains used in this study are detailed in Table 1. The only live attenuated vaccine strain currently in use in South Korea, AP-04203-P100, along with several previously isolated Korean wild-type strains, were used for SNP site analysis for differentiating between the wild-type and vaccine strains. The AP-04203-P100 and AP-04203 strains were employed for the development of the MAMA-PCR method. The propagation of the virus was carried out following established protocols [21]. Following a 5-day incubation at 37 °C, the viruses were harvested and passaged twice, and the viral stock was stored at −70 °C until further use [22]. The 50% embryo lethal dose (ELD_50_) was calculated according to previously described methods [23].

### 2.2. SNP Site Analysis and Primer Design

Five DHAV-3 wild strains from South Korea were selected, and their sequences were sourced from the NCBI GenBank database. Subsequently, the ClustalW tool integrated into the MegAlign software (DNAStar Lasergene, version 11) was employed for alignment. Primers DHAV-3-VP0-F (GATCCACACTGCCTGATAGG) and DHAV-3-VP0-R (CCGCCGTGGTTTCTTCTTACC) were designed based on the highly conserved region of the VP0 gene and used for partial VP0 gene sequencing of the vaccine strain AP-04203-P100. Nucleotide and amino acid polymorphisms within the VP0 gene that differentiate Korean wild-type strains from the vaccine strain were subsequently analyzed using the same sequence alignment method. The MAMA-PCR primers were designed based on the vaccine-specific SNP, which was incorporated into the 3′ end of the primers. To further enhance primer specificity, additional mismatches were introduced at the third-to-last and second-to-last positions of the 3′ end [25]. Specifically, the DHAV-3-MAMA-PCR-R1 primer includes an additional mismatch at the third-to-last position, while the DHAV-3-MAMA-PCR-R2 primer includes an additional mismatch at the second-to-last position of the 3′ end. The detection primers DHAV-3-pAF-new and DHAV-3-pAR-new were extended from previously published primers targeting the conserved 5′NCR region of DHAV-3 to enhance specificity [26]. The PCR primers were synthesized by Bioneer Corporation (Daejeon, Republic of Korea) (Table 2).

### 2.3. RNA Extraction and MAMA-PCR Reaction

RNA was extracted from allantoic fluid or tissue samples using the Gene-spin™ Viral DNA/RNA Extraction Kit (iNtRON, Daejeon, Republic of Korea), following the manufacturer’s instructions. RNA reverse transcription (RT) was performed according to a previously described protocol [27].

For the MAMA-PCR assay, we first compared the reaction efficiency of the DHAV-3-MAMA-R1 and DHAV-3-MAMA-R2 primers. Next, we optimized the primer concentrations of DHAV-3-pAF-new, DHAV-3-pAR-new, and DHAV-3-MAMA-R2 and the Mg^2+^ concentration for the reaction. Once the optimal reaction conditions were determined, adjustments were made to the annealing temperature, extension time, and cycle number in the PCR protocol. All PCR reactions were performed using the C1000 Touch™ Thermal Cycler (Bio-Rad, Hercules, CA, USA). The reaction mix, except for primers and water, was entirely composed of components from the Solg™ e-Taq DNA Polymerase set (Solgent, Daejeon, Republic of Korea).

### 2.4. Sensitivity and Specificity of MAMA-PCR

The sensitivity was evaluated by performing tenfold serial dilutions of wild-type strain samples in deionized water, followed by RNA extraction and reverse transcription for each dilution. The MAMA-PCR detection range for wild-type DHAV-3 was from 10^4.4^ to 10^1.4^ ELD_50_/mL. The vaccine strain was processed similarly, with a detection range of 10^2.5^ to 10^−2.5^ ELD_50_/mL.

In addition, to evaluate the specificity of the MAMA-PCR method, 11 common viral or bacterial pathogens in ducks including duck hepatitis A virus-1 (DHAV-1), avian influenza virus (AIV), duck enteritis virus (DEV), duck parvovirus (DPV), duck circovirus (DuCV), duck Tembusu virus (DTMUV), *Riemerella anatipestifer* (RA), *Pasteurella multocida* (PM), *Clostridium perfringens* (CP), *Mycoplasma gallisepticum* (MG), and *Mycoplasma synovialis* (MS) were used to evaluate potential cross-reactivity.

### 2.5. Detection of Clinical Samples

To assess the effectiveness of the developed MAMA-PCR method for detecting and differentiating wild-type and vaccine strains of DHAV-3 in clinical samples, specimens were collected from 89 farms located in major duck-rearing regions of South Korea (Jeollabuk-do, Jeollanam-do, Chungcheongbuk-do, Chungcheongnam-do, and Gyeonggi-do) between 2013 and 2022. Each farm provided 5 to 20 deceased dead or live ducklings suspected of having DHAV infections; most had exhibited clinical symptoms such as lethargy, ataxia, and opisthotonos. Post-necropsy examination revealed significant pathological findings, including hepatomegaly, hemorrhage, and congestion. Liver samples were pooled according to farm origin and stored at −70 °C. A 20% tissue homogenate was prepared in PBS with 1% antibiotic-antimycotic (Invitrogen, Carlsbad, CA, USA), centrifuged at 13,000 rpm for 10 min at 4 °C, and the supernatant was processed for RNA extraction and cDNA synthesis.

The accuracy of the developed MAMA-PCR method for detecting DHAV-3 in clinical samples was evaluated by comparing it with the previously described Korean DHAV-3 nested PCR detection method [6]. For PCR-positive samples, the VP0 gene was amplified and sequenced using the DHAV-3-VP0-F/R primers, and the SNP sites distinguishing wild-type and vaccine strains were analyzed. The sequencing results were compared with the differentiation results of the MAMA-PCR method to assess its ability to detect and differentiate wild-type and vaccine strains in clinical applications.

## 3. Results

### 3.1. Development of a MAMA-PCR Assay Based on a Single Mutation

**Figure 1:**
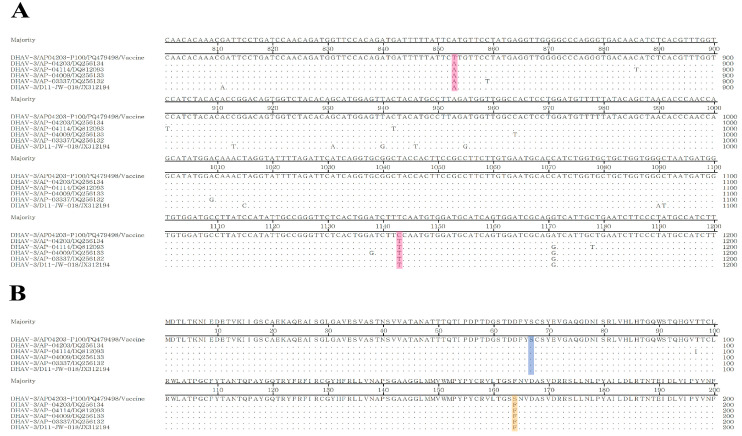
Comparison of the VP0 gene between the Korean DHAV-3 vaccine strain AP04203-P100 and wild-type strains. The nucleotide and corresponding amino acid sequences of the vaccine strain and four known Korean wild-type strains from NCBI were aligned and analyzed using the MegAlign program (DNAStar Lasergene, version 11). (**A**) Two distinguishing SNP sites were identified at positions 853 and 1143 in the VP0 gene (highlighted in red). (**B**) The SNP at position 853 does not alter the amino acid, with both vaccine and wild-type strains retaining serine (Ser, S), indicating a synonymous mutation (highlighted in blue). In contrast, the SNP at position 1143 results in an amino acid change from phenylalanine (Phe, F) to serine (Ser, S), indicating a non-synonymous mutation (highlighted in yellow).

The Korean DHAV-3 vaccine strain AP-04203-P100 exhibited nucleotide changes in the VP0 gene, specifically at positions 853 (A→T) and 1143 (T→C), as identified through multiple sequence alignment with wild-type strains (Figure 1A). Further analysis of the corresponding amino acid positions showed that despite the nucleotide mutation at position 853, it did not alter the amino acid sequence, which remained serine (Ser, S), indicating a synonymous mutation. However, the nucleotide mutation at position 1143 resulted in an amino acid change from phenylalanine (Phe, F) to serine (Ser, S), representing a non-synonymous mutation (Figure 1B). Consequently, the nucleotide at position 1143 was selected as the SNP target for the MAMA-PCR primer design. During the design of MAMA-R1 and MAMA-R2 primers, the 1143 nucleotide polymorphism was incorporated at the 3′ end of both primers. The key difference was that MAMA-R1 included an additional mismatch at the third-to-last position (T→A), while MAMA-R2 had an additional mismatch at the second-to-last position (G→A). MAMA-R2 produced fewer non-specific bands during amplification, demonstrating higher specificity, and was therefore selected for further method development.

The MAMA-PCR reaction system consisted of 2.5 μL of 10X e-Taq Reaction Buffer, 1.5 μL of 2 mM dNTP Mix, 0.25 μL of Solg™ e-Taq DNA Polymerase, 1.5 μL of forward primer DHAV-3-pAF-new (10 pmol), 0.3 μL of reverse primer DHAV-3-pA3R-new (10 pmol), 1 μL of DHAV-3-MAMA-R2 (10 pmol), and 15.95 μL of deionized water for a total volume of 25 μL. The PCR cycling conditions were as follows: initial denaturation at 94 °C for 5 min, followed by 35 cycles of denaturation at 94 °C for 1 min, annealing at 65 °C for 1 min, and extension at 72 °C for 40 s, with a final extension at 72 °C for 10 min. Amplification products were analyzed via 1.5% agarose gel electrophoresis.

**Figure 2:**
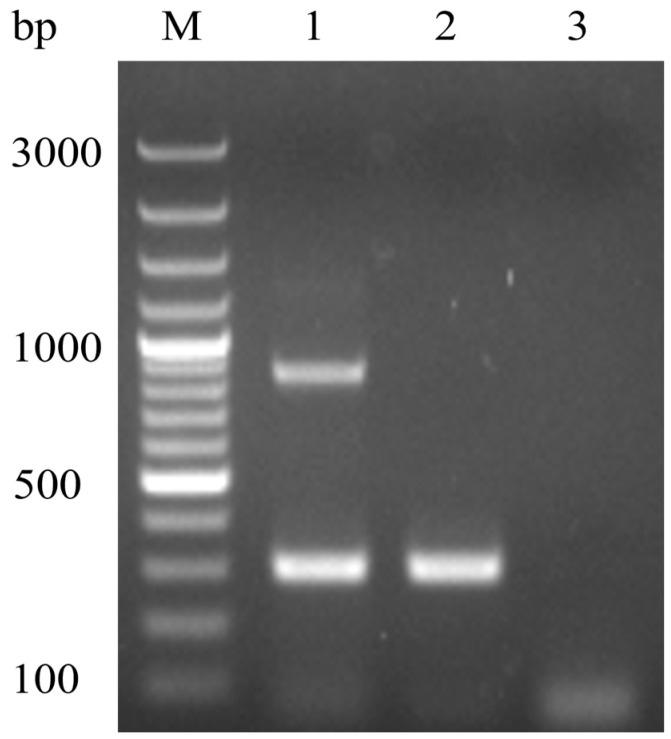
Agarose gel of MAMA PCR products. M: 100 bp DNA marker; Lane 1: vaccine strain showing bands at 870 bp and 297 bp; Lane 2: wild-type strain showing a single band at 297 bp; Lane 3: negative control, with no nonspecific bands observed.

The agarose gel electrophoresis results showed two distinct bands at 870 bp and 297 bp for the DHAV-3 vaccine strain, while only a single band at 297 bp was observed for the wild-type strain. No noticeable nonspecific bands were present, and no bands were observed in the negative control (Figure 2).

### 3.2. Validation of Sensitivity and Specificity of the MAMA-PCR Method

**Figure 3:**
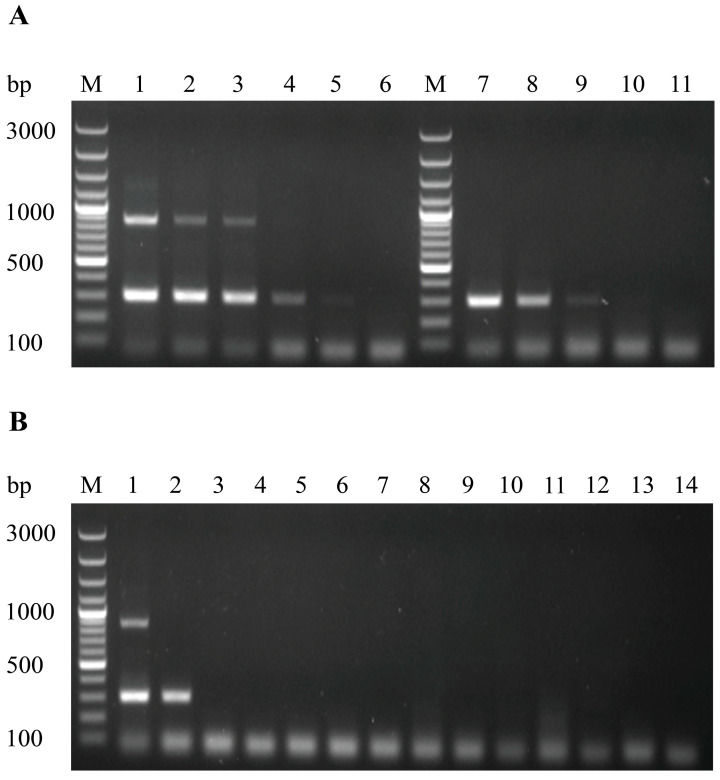
The sensitivity and specificity analysis of the MAMA-PCR method. (**A**) Sensitivity analysis results. M: 100 bp DNA Marker; Lanes 1~6: vaccine strain 10^2.5^~10^−2.5^ ELD_50_/mL (double bands at 870 bp and 297 bp were observed at 10^0.5^ ELD_50_/mL; only the 297 bp band was detected at 10^−0.5^ ELD_50_/mL, indicating the detection limit for the vaccine strain is 10^0.5^ ELD_50_/mL); Lanes 7~10: wild-type strain 10^4.4^~10^1.4^ ELD_50_/mL (a single 297 bp band was observed at 10^2.4^ ELD_50_/mL, and the band disappeared at 10^1.4^ ELD_50_/mL, indicating the detection limit for the wild-type strain is 10^2.4^ ELD_50_/mL); Lane 11: negative control. (**B**) Specificity analysis results. M: 100 bp DNA Marker; Lane 1: vaccine strain; Lane 2: wild-type strain; Lanes 3~13: DHAV-1, AIV, DEV, DPV, DuCV, DTMUV, RA, PM, CP, MG, MS; Lane 14: negative control. No amplification was observed for any of these 11 common duck pathogens.

To determine the sensitivity of the developed MAMA-PCR method for detecting and distinguishing between DHAV-3 wild-type and vaccine strains, cDNA samples from different dilutions of vaccine and wild-type strains were tested following tenfold serial dilutions. The results are shown in Figure 3A. At a concentration of 10^0.5^ ELD_50_/mL, distinct double bands at 870 bp and 297 bp were clearly observed, while at a concentration of 10^−0.5^ ELD_50_/mL, only the 297 bp band appeared.

For wild-type strain detection, a discernible single 297 bp band was still present at a concentration of 10^2.4^ ELD_50_/mL, but the band disappeared completely when the concentration decreased to 10^1.4^ ELD_50_/mL. Thus, the detection limit for the wild-type strain using this method is 10^2.4^ ELD_50_/mL.

When RNA or DNA extracted from DHAV-1, AIV, DEV, DPV, DuCV, DTMUV, RA, PM, CP, MG, and MS were used for specificity testing, no bands appeared at the target size, indicating no cross-reactivity with any of these 11 common duck pathogens (Figure 3B). These results demonstrate that the MAMA-PCR method designed for DHAV-3 exhibits high specificity.

### 3.3. Application in Clinical Samples

**Figure 4:**
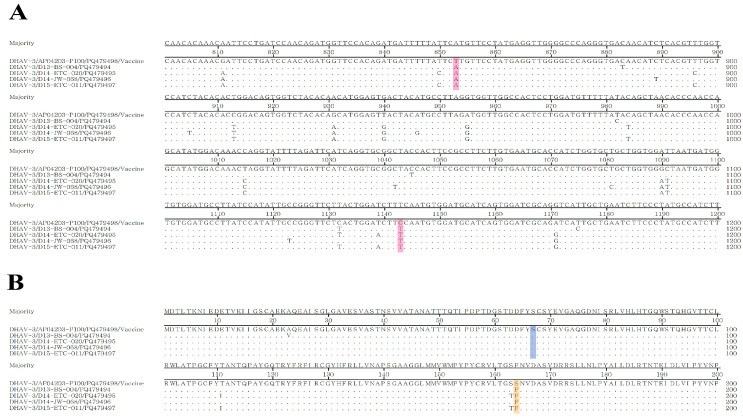
The comparison and analysis of nucleotide and amino acid sites between the Korean DHAV-3 vaccine strain AP04203-P100 and clinical positive samples. (**A**) Nucleotide site analysis. Differences were observed at nucleotide positions 853 and 1143 in the VP0 gene between the four clinical positive samples and the vaccine strain, as indicated by the red highlight. (**B**) Amino acid site analysis. The nucleotide variation at position 853 did not result in an amino acid change (highlighted in blue), while the nucleotide changes at position 1143 caused a substitution between serine (Ser, S) in the vaccine strain and phenylalanine (Phe, F) in the wild-type strain (highlighted in yellow). These findings are consistent with previous analyses comparing AP04203-P100 with NCBI wild-type strains, suggesting that the clinical samples represent wild-type strains.

**Figure 5:**
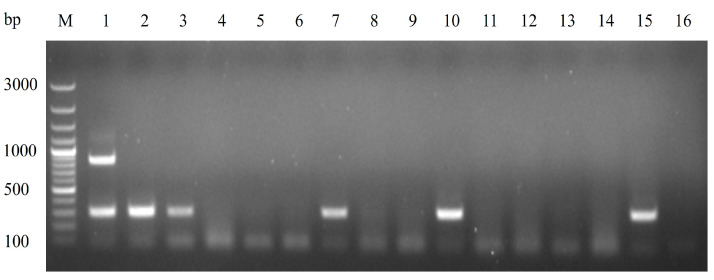
Detection results of MAMA-PCR on selected clinical samples. M: 100 bp DNA Marker. Lines 1~2: vaccine and wild-type strain; Lines 4~15: clinical samples; Lane 16: negative control. Clinical samples in Lanes 3, 7, 10, and 15 of D13-BS-004, D14-ETC-002, D14-JW-068, and D15-ETC-011 showed a single band, consistent with the wild-type result.

Using the MAMA-PCR approach described here, samples from 89 farms were analyzed, and four of these samples showed a distinct band, observed exclusively at 297 bp, indicating DHAV-3 wild-type strain infections. The findings were further confirmed by nested PCR, with the results fully consistent. Detailed information of the positive samples is presented in Table 3. Since nested PCR only serves as a detection method, the reliability of the MAMA-PCR differentiation results was further validated by amplifying and sequencing the VP0 gene of positive samples using DHAV-3-VP0-F/R primers. Analysis of SNPs at positions 853 and 1143 along with the corresponding amino acid variations confirmed that all samples from the four farms were DHAV-3 wild-type strains (Figure 4). The detection and differentiation results using the MAMA-PCR method were in complete agreement with the gold standard sequencing method, demonstrating the clinical applicability of the MAMA-PCR method (Figure 5).

## 4. Discussion

DHAV-3 is a significant pathogen in the duck industry, and live attenuated vaccines remain the most effective means of controlling its spread [10,11]. However, distinguishing vaccine strains from wild-type strains is critical for outbreak management, vaccination efficacy, and epidemiological surveillance [13,28,29]. Sequencing is currently the primary method for differentiating between these strains, but its application is limited in clinical settings due to its time inefficiency and high cost [17,30]. In response to this gap, our study developed a MAMA-PCR method targeting SNP variations in the VP0 gene of the AP-04203-P100 vaccine strain to provide a more rapid, accessible, and cost-effective alternative.

DHAV-3, a typical picornavirus, possesses a single-stranded, positive-sense RNA genome encoding structural proteins including VP0, VP1, and VP3, which play key roles in viral antigenicity and immunogenicity [14,31,32,33]. Mutations in these proteins are crucial for viral attenuation and form the basis for differentiating vaccine and wild-type strains. Among these, VP0 is a precursor protein and is located on the surface of the viral capsid [34]. Mutations in VP0 have been closely associated with the attenuation of virulence in various DHAV-3 strains. Furthermore, during the attenuation of field strains of DHAV-3, mutations such as Y164N and F164S were identified, which significantly reduced the pathogenicity of the virus and formed the basis for differentiating wild-type from vaccine strains [10,35]. The F164S mutation in the VP0 gene was a critical finding during the development of the Korean DHAV-3 AP-04203-P100 vaccine strain [10]. This mutation, caused by a T→C nucleotide substitution at position 1143 (F164S), was identified as a unique marker of the vaccine strain. While VP1 has traditionally been used to study the evolution of DHAV viruses due to its high genetic diversity, its rapid mutation rate makes it less suitable for diagnostic purposes. In contrast, VP0, which is more stable and associated with key attenuation mutations, provides a reliable target for differentiating between vaccine and wild-type strains. Given that VP0 mutations, including the change at position 1143 (F164S), are closely tied to the attenuation process, we selected VP0 as the target gene for the MAMA-PCR method. Therefore, to enable precise differentiation of vaccine strains from wild-type strains, the key mutation at position 1143 (F164S) in VP0 was successfully exploited to design the MAMA-PCR primers for detection.

The ability of the MAMA-PCR method to differentiate strains relies on mismatches introduced at the 3′ end of the primers, which prevent the amplification of non-matching templates [36]. Additionally, the dinucleotide mismatch strategy, in which extra mismatch bases are introduced at the 3′ end of primers, effectively minimizes the amplification of non-complementary primers [18,25]. By optimizing the reaction conditions, the MAMA-PCR system effectively distinguished vaccine strains from wild-type strains with high specificity in this study. The vaccine strains displayed two bands and the wild-type strains showed only one band. Our method demonstrated robust sensitivity, with detection limits as low as 10^2.4^ ELD_50_/mL for wild-type strains and 10^0.5^ ELD_50_/mL for vaccine strains. Compared with conventional DHAV detection methods, the developed MAMA-PCR method improves discriminatory power without compromising sensitivity [2]. This is particularly significant given the reduced replication capacity of attenuated vaccine strains, which results in lower viral titers in clinical samples than those of wild-type strains [35]. The increased sensitivity of our method enables reliable detection of low-titer vaccine strains, ensuring more effective monitoring of vaccinated flocks. Moreover, the specificity of the MAMA-PCR method was confirmed by testing it against 11 common viral and bacterial pathogens affecting ducks, including DHAV-1, AIV, DEV, DPV, DTMUV, DuCV, RA, PM, CP, MG, and MS. The absence of cross-reactivity confirmed the effectiveness of this method for diagnosing complex clinical infections.

In this study, the MAMA-PCR method was applied to 89 suspected duck hepatitis samples and identified four farms with DHAV-3 wild-type infections. The results were fully consistent with those obtained via nested PCR and confirmed via sequencing, which is the gold standard method [6,17]. This demonstrates that the developed MAMA-PCR method can diagnose DHAV-3 and differentiate between vaccine and wild-type strains within a few hours. This rapid detection capability is critical, particularly given the rapid onset and high mortality associated with DHAV-3 infections, enabling timely interventions to prevent the spread of the virus and reduce economic losses [1]. Moreover, the ability to distinguish infected flocks from vaccinated ones, especially in the early stages of infection or before clinical symptoms appear, facilitates more effective management and minimizes the risk of outbreaks [37]. Since there is currently only one DHAV-3 vaccine used in Korea, this method was specifically designed to differentiate the Korean vaccine strain. However, the simplicity of the MAMA-PCR design allows for quick adaptation to other vaccine strains should they be introduced in the future. By targeting specific SNP variations in different vaccine strains, the MAMA-PCR method can be rapidly modified, thus making it a flexible and valuable tool for differentiating DHAV-3 strains under varying vaccination protocols. This adaptability gives MAMA-PCR a distinct advantage, as it can be easily adjusted to accommodate evolving vaccination strategies and continuously support disease monitoring. This feature is especially valuable in resource-limited environments and when dealing with large-scale samples, where access to advanced sequencing technology may be restricted.

## 5. Conclusions

In conclusion, the MAMA-PCR assay demonstrated both high specificity and sensitivity, successfully detecting wild-type strains at 10^2.4^ ELD_50_/mL and vaccine strains at 10^0.5^ ELD_50_/mL. This rapid, one-step detection method outperforms traditional sequencing, making it highly suitable for clinical applications. Moreover, this method is also a valuable tool to accurately monitor vaccinated flocks and facilitate early detection of infections even before clinical symptoms appear, improving management of DHAV-3 outbreaks and minimizing economic losses in duck farming.

## Figures and Tables

**Table 1:** Virus strain information for sequence analysis.

Strain	Type	Origin	Accession Number	Reference
AP-04203-P100	Vaccine strain	South Korea	PQ479498	In this study
D13-BS-004	Wild strain		PQ479494	
D14-ETC-020			PQ479495	
D14-JW-068			PQ479496	
D15-ETC-011			PQ479497	
AP-04203			DQ256134	[2]
AP-03337			DQ256132	
AP-04009			DQ256133	
AP-04114			DQ812093	[24]
D11-JW-018			JX312194	[21]

**Table 2:** Primers used in this study for PCR amplification.

Primer	Sequences (5′-3′)	Target Gene	Wild-Type Strain Size (bp)	Vaccine Strains Size (bp)
DHAV-3-MAMA-R1	CGATCCACTGATGCATCCACATAGG	VP0+5′-NCR	297	870 and 297
DHAV-3-MAMA-R2	CGATCCACTGATGCATCCACATTAG			
DHAV-3-pAF-new	GGAGGTGGTGCTGAAATATTGCAAGC			
DHAV-3-pAR-new	GGCAGTGTGGATCAAAGGGGTTTTC			

**Table 3:** Summary of characteristics of four clinically positive samples.

Farm	Sample Type	Age (Days)	Anatomical Symptoms	Other Pathogen Diagnosis Results	Diagnostic Result
D13-BS-004	Dead duck	4	Hepatic hemorrhage; Renal hemorrhage; Intestinal hemorrhage; Splenomegaly with hemorrhage	AIV negative; *Salmonella* positive	DHAV-3 positive (wild strain); *S.* Typhimurium positive
D14-ETC-020	Dead duck	7	Renal hemorrhage; Thymic hemorrhage	AIV, DuCV, DEV, DPV, EDSV, RA, *Salmonella*, PM negative; APEC positive	DHAV-3 positive (wild strain); APEC positive
D14-JW-068	Dead duck	7	Hepatic hemorrhage; Intestinal hemorrhage	AIV, RA, *Salmonella*, APEC negative	DHAV-3 positive (wild strain)
D15-ETC-011	Dead duck	11	Hepatic degeneration; Renal hemorrhage; Uric acid deposition	AIV, DuCV, DEV, RA negative; *Salmonella*, APEC positive	DHAV-3 positive (wild strain); *S.* Typhimurium, APEC positive

Abbreviations: DHAV-3, duck hepatitis A virus 3; AIV, avian influenza virus; DuCV, duck circovirus; DEV, duck enteritis virus; DPV, duck parvovirus; EDSV, egg drop syndrome virus; RA, *Riemerella anatipstife*r; PM, *Pasteurella multocida*; APEC, avian pathogenic *Escherichia coli*; *S.* Typhimurium, *Salmonella* Typhimurium.

## Data Availability

The data presented in this study are available from the corresponding author on reasonable request.
